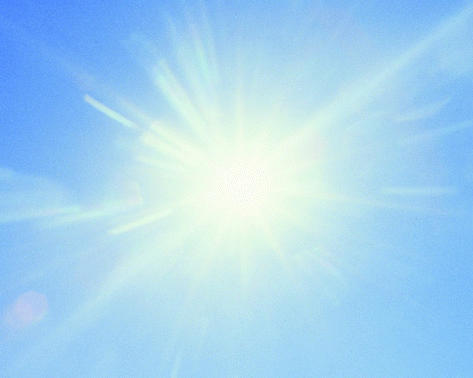# The Beat

**Published:** 2005-06

**Authors:** Erin E. Dooley

## Seaweed for Safety

Researchers from Oregon State University and Northeastern University have found that certain red seaweeds including *Portieria hornemannii* and *Acrosiphonia coalita* can detoxify organic pollutants such as TNT and polycyclic aromatic hydrocarbons 5–10 times faster than any known terrestrial plant. TNT is found at the sites of sunken warships, while polycyclic aromatic hydrocarbons are emitted from watercraft. The scientists, who presented their research at the 2005 annual meeting of the American Association for the Advancement of Science, see their discovery possibly playing a role in seafood safety, with marine seaweeds being planted around aquaculture beds to protect oysters, clams, and other bioaccumulators from contamination.

## Nations’ Environmental Efforts Ranked

In January 2005 the second Environmental Sustainability Index was released, ranking 146 nations on their environmental stewardship efforts. Prepared by researchers at Yale and Columbia, the index is based on 75 measures, including hazardous waste generation, pesticide consumption, participation in international environmental agreements, and carbon emissions. The first index, which came out in 2002, was a wakeup call for countries, inspiring some to improve their performance. South Korea, for example, moved up 13 spots between the first and second indexes.

Finland, Norway, and Uruguay took the top three positions, while the United States ranked 45th. The lowest-ranking country was North Korea. The report cited a significant correlation between higher ranking and countries having open political systems and effective governments.

## Sustainable Wildcrafting in Nepal

In Nepal, approximately 15,000 tons of medicinal plants are collected for export each year by villagers who often receive less than a living wage for their work and are encouraged by unscrupulous buyers to strip plant supplies. A coalition of Nepalese and U.S. product buyers, advocacy groups, and donors was set up in 2002 to promote sustainable collection among villagers and responsible buying among western purchasers, with certification as one incentive. These efforts are paying off: in January 2005 the Federation of Community Forestry Users, Nepal, received certification from the Rainforest Alliance for its handmade paper and herbal products. The federation’s members manage community forests by sustainable principles and supply wildcrafted ingredients to the international herbal, medicinal, and natural products industries.

## Obesity Cuts Longevity

The surge of obesity, especially among children and adolescents, could shorten life expectancy in the United States by 2–5 years, reversing the steady rise in longevity of the past two centuries, says a data analysis published in the 17 March 2005 *New England Journal of Medicine*. The predictions are based on data from the Third National Health and Nutrition Examination Survey and previously published reports on estimated years of life lost from obesity. Current trends indicate that rates of obesity will continue to rise, and that ever-younger age groups will be affected. The surge in obesity has already triggered a sharp rise in type 2 (“adult-onset”) diabetes mellitus in children.

## EU Holds Firm on REACH

On 4 April 2005 the European Commission announced its plan to introduce its controversial Registration, Evaluation, and Authorisation of Chemicals (REACH) policy for consideration by the European Parliament. The policy calls for the chemical industry to provide safety data for 30,000 chemicals. Business leaders, citing costs for testing, had urged the commission to require rigorous testing only for the 4,000–5,000 chemicals that pose the greatest risks. An impact study published 27 April 2005 did confirm that the policy could be pricey for businesses. But EU environment commissioner Stavros Dimas said he is convinced that the plan strikes the proper balance between protecting environmental and human health and protecting business interests. REACH was first proposed in October 2003 [see “REACHing for Chemical Safety,” *EHP* 111:A766–A769 (2003)]. A final decision is expected in early 2006.

## CFCs: A Dying Breed

China and Venezuela have pledged to phase out the use and production of chlorofluorocarbons (CFCs) by the end of 2007, two years earlier than required by the Montréal Protocol on Substances that Deplete the Ozone Layer. A total of US$26.5 million from the protocol’s Multilateral Fund has been allocated to finance the phaseouts. With these pledges, production of more than 100,000 tons of CFCs will be eliminated each year, and the use of CFCs in developing countries will end. China is the world’s largest producer and consumer of CFCs, which are used as coolants, solvents, and propellants.

Protection against further degradation of the ozone layer should prevent millions of cases of skin cancer and cataracts resulting from harmful ultraviolet rays reaching the Earth’s surface. The phaseout also means fewer emissions contributing to global warming.

## Figures and Tables

**Figure:**
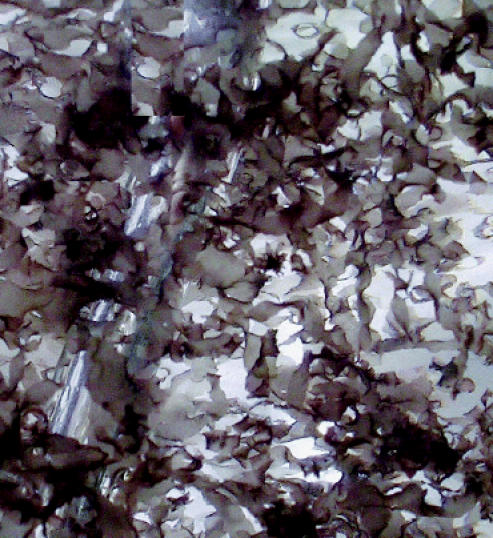


**Figure:**
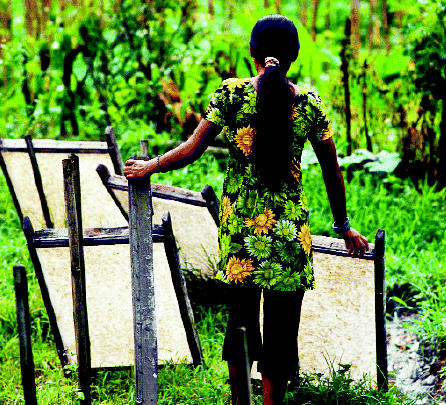


**Figure:**
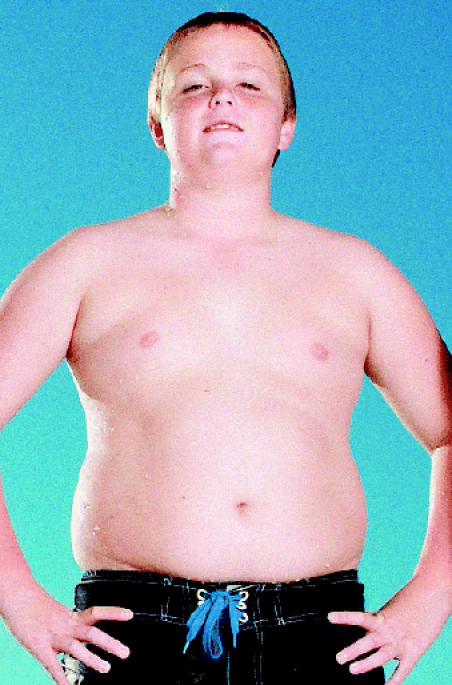


**Figure:**